# Investigating the relationships between social capital, chronic health conditions and health status among Australian adults: findings from an Australian national cohort survey

**DOI:** 10.1186/s12889-020-8370-0

**Published:** 2020-03-14

**Authors:** Jeong Kyu Lee, Lavinia Lin, Christopher Magee

**Affiliations:** 1 Saw Swee Hock School of Public Health, National University of Singapore and National University Health System, Singapore, Singapore; 2 School of Psychology, University of Wollongong, Wollongong, Australia

**Keywords:** Social capital, Health status, Chronic Health conditions, Cohort data

## Abstract

**Background:**

Social capital is a collective attribute of communities that determines health and well-being of populations. The collective resources in a high social capital community have been reported to result in better health outcomes. While evidence supports the links between social capital and various health outcomes, it is not clear about underlying mechanisms connecting multiple dimensions of social capital to health.

**Methods:**

Using the two-wave data from a nationally representative cohort study of Australian adults (*N* = 16,637), this study examined the effects of two dimensions of social capital (i.e., structural and cognitive social capital) on physical and mental health in the Australian adult population. Based on prior literature and theoretical reasoning, it was anticipated that the structural and cognitive social capital would influence self-assessed health status (physical and mental health). Additionally, these two dimensions of social capital were hypothesized to moderate the relationships between chronic health conditions and these two aspects of health status.

**Results:**

Analyses showed that the effects of chronic health conditions on mental health status were moderated by the structural social capital (*β* = .652, *SE* = .249, *p* = .009). Additionally, it was found that perceived community cohesion was predictive of mental health (*β* = .295, *SE* = .103, *p* = .004). Our analysis also indicated that perceptions of disadvantaged neighbourhood environment contributed to poorer mental health status (*β* = −.461, *SE* = .144, *p* = .001). However, none of the social capital variables significantly predicted physical health status.

**Conclusions:**

Findings suggest that the structural dimension of social capital would function as a buffer against the malicious effects of chronic health conditions, impairments and disabilities. Specifically, community participation (structural social capital) is indispensable to develop an effective community-based program to improve health and well-being of those with chronic health conditions or disabilities, as increasing active participation may generate beneficial effects in this vulnerable population. Subjective perceptions about communities can also play an important role in improving better health outcomes. Further research is needed to examine underlying mechanisms linking the multiple dimensions of social capital to health outcomes among individuals who are vulnerable to external stressors.

## Background

The impact of social environmental factors on health and well-being has been widely studied over decades [1]. Since individuals’ behaviours and their social relations are embedded in neighbourhoods and communities, the concept of social capital provides a valuable conceptual perspective to understand how social environment influences health outcomes and behaviours [2, 3]. Social capital is an important determinant of health and overall well-being [4]. Despite increasing acknowledgement, social capital suffers from a lack of consensus on its operationalization and measurement as the definitions and concepts are malleable depending on different contexts [5].

In the fields of public health and social epidemiology, Robert Putnam’s definition of social capital has been widely utilised [6]. Putnam conceived of social capital as a collective attribute of communities and societies, and it is commonly characterised by social cohesion, trust, norms of reciprocity and density of membership [7]. Building on his conceptualization [8–11], the current study focused on two distinct dimensions of social capital: *structural* (i.e., what people do) and *cognitive* (i.e., what people feel) components. The structural component relates to the composition, extent and intensity of participation in the community, and memberships of social groups and organisations [11–13]. The cognitive component of social capital, on the other hand, refers to subjective perceptions about the community resulting from participation [2, 14]. In this study, the cognitive component was operationalised by perceptions of community cohesion encompassing neighbourhood trust and belongingness [14, 15].

### Social capital and Health

Numerous studies have found significant associations between social capital and various health behaviours and outcomes, such as self-assessed health [16–18], mental health and well-being [12, 19], psychological distress [13], cardiovascular and cancer mortality [20], vegetable and fruit consumption [21], physical activity [22], and smoking cessation [23–25]. It has been suggested that social capital can influence health through various mechanisms [2]. Community participation (structural social capital) is known to improve health, as it acts as a conduit for the transmission of knowledge [26]. It can also strengthen behavioural norms or adoption of related behaviours [27, 28]. Subjective perceptions about communities (cognitive social capital) also play an important role in developing and maintaining positive health status [29]. One explanation is that neighbours and other members of the community could act as an important source of trust and support if the community is cohesive, building strong connections among the community members [30, 31]. A recent study reinforced this, finding that neighbourhood cohesion is associated with an increased likelihood of preventive healthcare use and accessibility [32].

Whereas social capital and other community resources (e.g., social support) facilitate better health outcomes, there are some neighbourhood characteristics worsening community health and well-being. Previous studies have suggested that perceived neighbourhood climate such as violence, noise, traffic, and vandalism, may have a negative impact on health and contribute to serious chronic conditions [33, 34]. Hence, this study also looked into the influence of perceptions about neighbourhood social climate (e.g., noise, violence, burglary and theft), in addition to the effects of social capital on health status.

### Chronic Health conditions in Australia

Chronic health conditions including impairments and disabilities are an emerging public health concern in Australia. Approximately half of the population reported that they have at least one prominent chronic conditions (e.g., arthritis, asthma, back pain, cancer, cardiovascular disease, chronic obstructive pulmonary disease, diabetes or mental health conditions), and nearly a quarter of all Australian (23%) and 60% of those aged over 65 years had two or more chronic conditions [35]. The prevalence of comorbidities is apparently increasing in Australia due to the older age of the population [36, 37]. Those with chronic health conditions are associated with poor health outcomes that may result in lower quality of life, functional decline, and shorter life expectancy [38, 39]. Some risk factors for chronic conditions include behavioural determinants, such as alcohol use, smoking and poor nutrition and diet, as well as social and economic determinants, which influence individual decisions about their lifestyle [35]. However, it is unclear whether social capital can be a buffer to the negative effect of chronic health conditions including impairments and disabilities.

In addition to the direct effects of social capital, evidence suggests that social capital can buffer against potentially negative consequences of various strains such as poverty, job loss, and retirement [40, 41], and negative influence of external stressors on health and related outcomes [42, 43]. In particular, Anwar and colleagues have recently explored a modifying role of social capital in the longitudinal effect of disability onset on mental health using a sophisticated analytic technique. Their findings showed that social capital was beneficial for individuals who had poorer mental health status before their acquisition of disability [44]. With all these evidence, it is reasonable to anticipate that social capital would buffer the influence of chronic health conditions (including impairments and disabilities) on health status (physical and mental health). Challenges lie in linking the concept of social capital to chronic health conditions that continues to strain the economies of many countries. By using the concept of social capital, the underlying problem surrounding the prevention and treatment of hypertension and diabetes, for instance can be better understood [45–47].

The current study, therefore, extended previous research by treating the multiple dimensions of social capital as potential moderators on the relationship between chronic health conditions and health status (physical health and mental health) in the Australia adult population. Using a nationally representative cohort data, this study aimed to: 1) examine the effects of the structural and cognitive components of social capital on self-reported physical and mental health status among Australian adults (i.e., direct effect hypothesis); and 2) assess whether the two components of social capital moderate the relationship between chronic health conditions and their health status (i.e., buffering effect hypothesis).

## Methods

This study utilised data from the Household, Income and Labour Dynamics in Australia (HILDA) Survey, a nationally representative longitudinal panel survey of Australian households established in 2001 [48]. Study samples were limited to adults aged 18 years and older (*N* = 16,637). The data were collected from Waves 14 and 15 (July 2014 to Feb 2016), which are referred to as *Time 1* and *Time 2* respectively in this paper.

The survey is administered annually using a combination of face-to-face interviews and self-completion questionnaire to collect information on social, demographic, health and economic conditions [49, 50]. The sampling unit of the HILDA Survey is household, following the definition of the Australian Bureau of Statistics (ABS). Detailed information about the HILDA methodology can be found at https://melbourneinstitute.unimelb.edu.au/hilda/for-data-users/user-manuals. The National University of Singapore’s Institutional Review Board exempted the study from ethics review as the data were de-identified by the Melbourne Institute and the HILDA operations team.

### Measures

#### Structural social capital (Time 1)

Items measuring levels of community participation were derived from the Australian Community Participation Questionnaire, which has been validated in previous report [15]. Following Berry and Welsh’s approach [12], a seven-item measure was used to assess individual levels of participation. These items include: 1) volunteering to work on boards or committees; 2) attending religious services; 3) organizing community activities; 4) getting involved in political activities; 5) attending community events; 6) giving money to charity; and 7) getting in touch with a local politician or councilor. The intensity of structural social capital were assessed using a six-point scale ranging from 1 (never) to 6 (very often). A higher mean score indicated a greater level of community participation. Cronbach’s alpha coefficient for the structural social capital measure was 0.75.

#### Cognitive social capital (Time 1)

Perceptions of community cohesion were assessed using a five-item measure rated on a seven-point ranging from 1 (strongly disagree) to 7 (strongly agree) [15]. Items include: “This is a close-knit neighbourhood,” “People around here are willing to help their neighbours,” “People in this neighbourhood can be trusted,” “People in this neighbourhood generally do not get along with each other,” and “People in this neighbourhood generally do not share the same values.” The last two items were reverse-coded before computing an average score value. A higher mean score indicated stronger perceptions of cohesion. Cronbach’s alpha coefficient for this measure was 0.78.

#### Neighbourhood social climate (Time 1)

Neighbourhood social climate were assessed using an eight-item measure on a five-point scale ranging from 1 (never happen) to 5 (very common) [51]. The neighbourhood characteristics included: 1) traffic noise, 2) noise from airplanes, trains, industry, 3) homes and gardens in bad condition, 4) rubbish and littering lying around, 5) teenagers handing around on the street, 6) people being hostile and aggressive, 7) vandalism and deliberate damage to property, and 8) burglary and theft. A higher mean score indicated a poorer quality of neighbourhood social environment. Cronbach’s alpha for the measure was 0.86.

#### *Health status* (Times 1 and 2)

Physical and mental health status (primary outcomes of the study) were evaluated at Times 1 and 2 using the Short-Form Health Survey (SF-36) scale, which is widely used to assess health and functioning in both clinical and non-clinical samples [52, 53]. This scale consists of 36 items to calculate eight subscales of health status: physical functioning, role-physical, bodily pain, general health, vitality, social functioning, role-emotional, and mental health. Following the recommendation by the SF-36 Survey designers [53], we computed the norm-based scoring of two component summary measures: Physical Component Score (PCS) and Mental Component Score (MCS). All eight subscales were first standardized using a linear transformation. The four subscales (physical functioning, role-physical, bodily pain, general health) and the other four subscales (vitality, social functioning, role-emotional, mental health) were then added up to form the two summary scores for physical and mental health status: PCS and MCS. We produced the two summary scores using the Australian population norms for the transformed scores, which were derived from the Australian National Health Survey [54]. Higher scores of the summary measures indicated better health status in the two domains of health – physical health and mental health.

#### Chronic health conditions, impairments, and disabilities (Time 1)

Participants were asked to report whether they had any of the following long-term health condition, impairment or disability restricting their everyday activities for 6 months or more. Conditions included: 1) sight problems, 2) hearing problems, 3) speech problems, 4) black outs, fits or loss of consciousness, 5) difficulty of learning/understanding, 6) limited use of arms or fingers, 7) difficulty gripping things, 8) limited use of feet or legs, 9) nervous or emotional condition, 10) any condition restring physical activity or work, 11) any disfigurement or deformity, 12) any mental illness, shortness of breath, 13) chronic or recurring pain, long-term effects as a result of head injury stroke, or other brain damage, 14) long-term condition or alignment still restrictive after being treated, and 15) other long-term conditions such as arthritis, asthma, heart disease, Alzheimer’s disease and dementia. A show card listing examples of chronic health conditions were presented as a prompt for participants. A dichotomised variable was constructed to identify those reported of chronic health conditions, impairments and disabilities in the past 6 months and those who did not report any health conditions.

#### Lifestyle related factors (Time 1)

Smoking consumption, drinking consumption, physical activity, and body mass index (BMI) were included. Smoking and drinking consumption were dichotomised as “yes” and “no” to identify participants’ current status of smoking and drinking consumption. Levels of physical activity were assessed using an eight-point scale (0–7). They were recoded into four categories: None, 1–2 times, 3–6 times, and Every day. BMI was calculated based on participants’ self-reported heights and weights, and those who provided insufficient or implausible information on their heights and weights were excluded from the calculation. Following the Australian Bureau of Statistics’ criteria [55], we recoded the BMI data into four categories: underweight (BMI < 18.5), normal weight (18.5–24.9), overweight (BMI 25–29.9), and obese (BMI ≥ 30).

#### Socio-demographic characteristics (Time 1)

The following socio-demographics were included: 1) age range (recoded as “18 to 24,” “25 to 44,” “45 to 64,” “65 and above”), 2) gender (male vs. female), 3) educational attainment (recoded as “bachelor degree or above,” “polytechnic diploma and certificate,” “year 12,” “year 11 or less”), and 4) country of birth (“Australia-born” vs. “foreign-born”).

### Statistical analysis

Data were analysed using *Mplus* Version 6.11 [56]. Statistical analyses (bivariate and multivariate) were performed to assess relationships between socio-demographics, social capital and health status among adults with and without chronic health condition, impairment or disability. Prior to regression analyses, preliminary tests for normality and multicollinearity were conducted. Results showed that data met the assumptions of normality and the scores of the variance inflation factor (*VIF*) ranged from 1.05 to 3.36, indicating low to moderate correlations among the variables used in the regression models.

Two sets of multiple linear regression models (main analysis) were built to assess the effects of social capital on the two summary measures of SF-36 (Model I predicting physical health status [PCS] and Model II predicting mental health status [MCS]), controlling for socio-demographic characteristics, lifestyle factors and the two summary scores at baseline. All the parameters were estimated using the maximum likelihood estimation method with robust standard errors. The analyses yielded adjusted regression weights (β) with standard errors (*SE*). The two regression analyses (Models I and II) were performed by regressing the two outcome variables – PCS and MCS on potential predictor variables, including the two components of social capital (i.e., community participation and perceived cohesion). The two summary scores, PCS and MCS at Time 2, were included in the regression models as the outcome variables and the two summary scores at Time 1 were used as covariates to adjust change over time. To test the moderation effects of the two dimensions of social capital on the relationship between chronic health conditions and health status, two interaction terms (community participation × chronic health conditions and perceived cohesion × chronic health conditions) were created and included in the regression models.

Statistical significance was assessed at .05 alpha level. Proportions of missing on the variables used in the analyses ranged from 0 to 22.9%. The full information maximum likelihood (FIML) estimation is known as a reliable missing data technique providing unbiased estimates of missing parameters in large samples, while retaining natural variability in the data [57]; thus, missing data were accommodated using the FIML method [58].

## Results

### Descriptive statistics

Table 1 displays the descriptive statistics for the complete samples (*N* = 16,637) and the two subgroups (i.e., those with and without chronic health conditions). The mean age of participants was 56.37 years (*SD* = 18.79 years), and 52.6% were females. About a quarter of them (25.8%) obtained a bachelor or postgraduate degree (e.g., graduate certificate, master and PhD). Approximately 30% (*n* = 4927) of the participants reported that they experienced one or more chronic health condition lasting 6 months or more. Details of the long-term health condition, impairment, and disability are presented in Table 1.

**Table 1:** Summary of the univariate and bivariate statistics

Variables	Full sample	Chronic condition(s)	No chronic condition	*p*-value
	N (%)	N (%)	N (%)	
Gender				0.004
Male	7887 (47.4)	2252 (45.7)	5635 (48.1)	
Female	8750 (52.6)	2675 (54.3)	6075 (51.9)	
Age				< 0.001
18 to 24	2333 (14.0)	358 (7.3)	1975 (16.9)	
25 to 44	5907 (35.5)	987 (20.0)	4920 (42.0)	
45 to 64	5319 (32.0)	1749 (35.5)	3570 (30.5)	
65 and older	3078 (18.5)	1833 (37.2)	1245 (10.6)	
Country of birth				0.004
Australia-born	12,919 (77.7)	3756 (76.2)	9163 (78.3)	
Foreign-born	3713 (22.3)	1170 (23.8)	2543 (21.7)	
Educational level				< 0.001
Year 11 and below	4187 (25.2)	1938 (39.4)	2249 (19.2)	
Year 12 and equivalent	2658 (16.0)	593 (12.1)	2065 (17.6)	
Diploma and certificate	5496 (33.1)	1566(31.8)	3930 (33.6)	
Bachelor’s and above	4285 (25.8)	822 (16.7)	3463 (29.6)	
Smoking status				< 0.001
Yes	2706 (18.4)	906 (20.7)	1800 (17.4)	
No	12,012 (81.6)	3473 (79.3)	8539 (82.6)	
Drinking status				< 0.001
Yes	12,097 (82.3)	3269 (74.7)	8828 (85.6)	
No	2594 (17.7)	1108 (25.3)	1486 (14.4)	
Physical activity (per week)				< 0.001
None	1739 (11.8)	990 (22.4)	749 (7.2)	
1 to 2 times	5926 (40.1)	1894 (38.4)	4232 (40.8)	
3 to 6 times	5427 (36.7)	1313 (29.8)	4114 (39.7)	
Everyday	1680 (11.4)	415 (9.4)	1265 (12.2)	
Body mass index (BMI)				< 0.001
Underweight	308 (2.2)	98 (2.4)	210 (2.1)	
Normal weight	5396 (38.3)	1210 (29.2)	4186 (42.1)	
Overweight	4970 (35.3)	1459 (35.2)	3511 (35.3)	
Obese	3416 (24.2)	1374 (33.2)	2042 (20.5)	
Chronic health condition, impairment and disability		N.A.	N.A.	N.A.
Sight problem	469 (9.5)			
Hearing problem	869 (17.6)			
Speech problem	84 (1.7)			
Blackouts, fits or loss of consciousness	149 (3.0)			
Difficulty learning	247 (5.0)			
Limited use of arms/fingers	611 (12.4)			
Difficulty gripping things	559 (11.3)			
Limited use of feet/legs	961 (19.5)			
Nervous or emotional condition	723 (14.7)			
Condition restricting physical activity	1790 (36.3)			
Disfigurement/deformity	102 (2.1)			
Mental illness	400 (8.1)			
Shortness of breath	626 (12.7)			
Chronic or recurring pain	1329 (27.0)			
Head injury or other brain damage	209 (4.2)			
Long-term condition still restrictive after treatment	1403 (28.5)			
Other long-term conditions	2169 (44.0)			
Physical health status (PCS) M (SE)	48.24 (12.14)	37.42 (13.59)	52.75 (7.94)	< 0.001
Mental health status (MCS) M (SE)	49.49 (12.25)	42.73 (14.14)	52.34 (10.09)	< 0.001
Community participation M (SE)	2.32 (0.81)	2.33 (0.84)	2.31 (0.80)	0.371
Perceived community cohesion M (SE)	4.66 (1.06)	4.61 (1.13)	4.68 (1.03)	0.001
Neighbourdhood social climate M (SE)	2.45 (0.69)	2.48 (0.73)	2.44 (0.70)	0.002

Note. *M* Mean, *SE* Standard error

Pearson’s Chi-square tests were used for categorical variables, while independent-sample t-tests were used for continuous variables

Significance levels for the bivariate analyses (chronic conditions vs. no chronic condition)

A series of bivariate analyses were conducted to assess differences in socio-demographic characteristics, lifestyle behaviours, social capital measures and self-assessed health status (SF-36) between those with and without chronic health conditions (Table 1). The analyses indicated that those with chronic health conditions were more likely to be female, older and less educated. The summary scores of the SF-36 were significantly lower among those who had chronic health conditions. Compared to those with chronic health conditions, participants who did not have chronic health conditions reported significantly higher level of perceived community cohesion. However, there was no significant difference in the structural social capital (i.e., community participation) between the two subgroups.

### Main analysis (multiple linear regression)

Table 2 presents the results of the first regression analysis (Model I) predicting physical health status (PCS) from the two dimensions of social capital, neighbourhood social climate, lifestyle and socio-demographic factors. As indicated in the Model I, there were no significant interactions of the two components of social capital and chronic health conditions on the physical health status (PCS) at .05 alpha level. In addition, the analysis did not find direct effects of the two dimensions of social capital on PCS. Chronic health conditions were significantly and inversely associated with the outcome variable (PCS) (*β* = − 3.539, *SE* = .224, *p* < .001).

**Table 2:** Multiple linear regression analyses for predictors of self-reported health status (PCS and MCS at Time 2)

Parameters	DV: PCS (R^2^ = .635)			DV: MCS (R^2^ = .515)		
	Unst. (St.)	SE	*p*-value	Unst. (St.)	SE	*p*-value
Gender (reference category: Female)						
Male	0.600 (0.025)	.146	<.001	0.771 (0.032)	.170	<.001
Age (reference category: 65 and above)						
18 to 24	3.239 (0.087)	.257	<.001	−1.938 (−0.051)	.370	<.001
25 to 44	3.191 (0.128)	.204	<.001	−1.198 (−0.047)	.282	<.001
45 to 64	1.973 (0.080)	.191	<.001	−0.220 (− 0.009)	.245	.369
Country of birth (reference category: Australia-born)						
Foreign-born	0.023 (0.001)	.177	.896	− 0.082 (− 0.003)	.206	.689
Education levels (reference category: Year 11 and below)						
Year 12 and equivalent	0.581 (0.022)	.223	.009	−0.359 (− 0.013)	.252	.154
Polytechnic diploma and certificate	0.080 (0.003)	.213	.707	−0.538 (− 0.021)	.241	.026
Bachelor degree and above	0.338 (0.010)	.255	.186	−0.339 (− 0.010)	.304	.265
Smoking status (reference category: No)						
Yes	−0.360 (− 0.011)	.290	.085	−1.222 (− 0.038)	.261	<.001
Drinking status (reference category: No)						
Yes	0.384 (0.012)	.212	.070	0.593 (0.018)	.248	.017
Physical activity per week (reference category: None)						
1–2 times	1.278 (0.053)	.286	<.001	−0.067 (−0.003)	.335	.841
3–6 times	1.473 (0.060)	.292	<.001	0.308 (0.012)	.341	.367
Everyday	1.107 (0.030)	.349	.002	0.356 (0.009)	.390	.361
BMI (reference category: Obese)						
Underweight	−0.313 (−0.004)	.614	.611	−0.178 (− 0.002)	.750	.813
Normal weight	1.456 (0.060)	.199	<.001	0.484 (0.019)	.231	.037
Overweight	1.095 (0.044)	.198	<.001	0.192 (0.008)	.228	.400
Physical health status (PCS at Time 1)	0.592 (0.585)	.012	<.001	0.117 (0.114)	.013	<.001
Mental health status (MCS at Time 1)	0.084 (0.083)	.010	<.001	0.596 (0.584)	.012	<.001
Chronic health condition, impairment and disability (reference category: No)						
Yes	−3.539 (−0.134)	.224	<.001	−1.719 (−0.064)	.254	<.001
Community participation	−0.009 (− 0.001)	.108	.933	0.153 (0.010)	.121	.206
Perceived community cohesion	0.066 (0.006)	.088	.449	0.295 (0.026)	.103	.004
Neighbourdhood social climate	−0.020 (−0.001)	.121	.872	−0.461 (− 0.026)	.144	.001
Community participation × chronic health conditions	−0.270 (− 0.010)	.220	.219	0.652 (0.024)	.249	.009
Perceived community cohesion × chronic health conditions	0.261 (0.013)	.170	.125	0.237 (0.012)	.202	.240

Note. *Unst.* Unstandardised regression weight, *St.* Standardised regression weight, *SE* Standard error. Gender, age, country of birth, education, smoking status, drinking status, physical activity, BMI, and chronic health conditions are dummy-coded variables

**Fig. 1:**
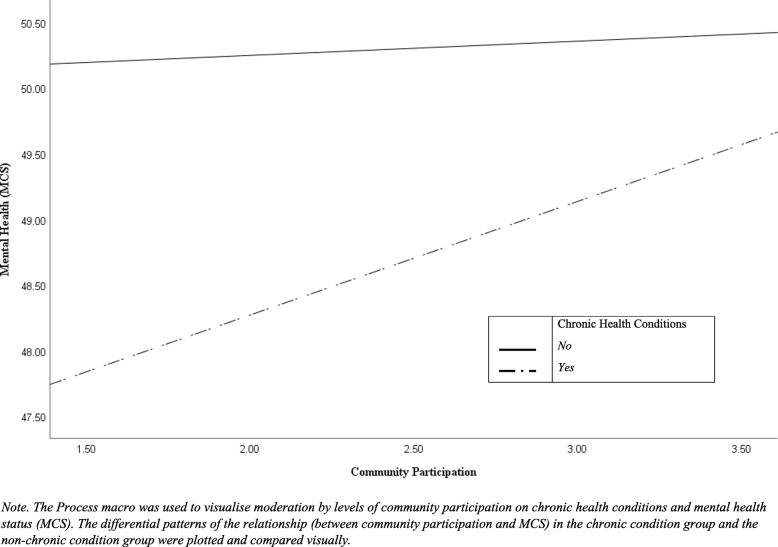
Moderation by levels of community participation on chronic health conditions and mental health status

The second regression analysis (Model II) was carried out to predict MCS using the same set of variables used in the Model I. The regression analysis revealed a significant moderation effect of community participation on the relationship between chronic health conditions and MCS, such that participants with chronic health conditions were more likely to report better mental health status as they engaged more often in the community (*β* = .652, *SE* = .249, *p* = .009) (see Fig. 1 for visual summary). While the interaction of perceived cohesion and chronic health conditions had no significant effect on MCS, there was a direct effect of perceived cohesion on the outcome variable, meaning that those who perceived a higher level of cohesion were more likely to report better mental health status (*β* = .295, *SE* = .103, *p* = .004). Our analysis also indicated that perceptions of disadvantaged neighbourhood environment contributed to poorer mental health status due to the significant and inverse relationship with MCS (*β* = −.461, *SE* = .144, *p* = .001).

Smoking status and drinking status were found to be significantly associated with MCS (Model II). Compared with those who were obese (BMI ≥ 30), participants who had normal weight were more likely to report better physical health and mental health status. Whereas levels of physical activity were significantly predictive of MCS, those who participated in physical activity at least once in a week reported better status of physical health (PCS) than those who were not participated in physical activity. Some of the socio-demographic factors introduced to the regression models significantly predicted self-reported health status. Gender and age were significantly associated with both PCS and MCS, such that those who were male and younger were more likely to report better status of physical and mental health; however, country of birth did not significantly influence any of the outcome variables.

## Discussion

The current study provided new evidence on the differential effects of the multiple components of social capital (i.e., community participation and perceived cohesion) on the two primary aspects of health status in the Australian adult population. This is an important area of research in public health, given that individual-level health outcomes are determined by social environmental factors including community and neighbourhood characteristics. This study built on prior research demonstrating that social capital is beneficial for a range of health related outcomes and behaviours [19]. Our study shows that the two dimension of social capital can play a crucial role in improving community health and well-being based on the findings on the direct relationships between the two components of social capital and health status. In addition, the study examined the buffering effects of social capital by testing the moderation on the relationship between chronic health conditions and the outcome variables. While a recent study [44] has suggested the modifying effect of social capital on the relationship between disability onset and mental health, the current study reported novel results of the moderation effects of social capital on individuals’ health outcomes. In this study, the buffering effect hypothesis was tested with rigorous conceptualisation of the multiple elements of social capital and the longitudinal research design.

Community participation is an indispensable attribute of the structural social capital [13]. Our notable findings showed that the beneficial effects of community participation on mental health were more pronounced among individuals with chronic health condition, impairment or disability. Chronic health conditions are a long-term stressor contributing to poor mental health and quality of life [59, 60]. Those with chronic health conditions are vulnerable to strains as these health conditions confer difficulties and troubles in many aspects of their life. The increasing prevalence of chronic conditions and comorbidities as well as aging populations have placed a great burden on individuals, communities and health care services in Australia [36, 37]. Based on our findings, it may be crucial to posit a community empowerment approach through integrating health and social services, promoting community partnerships and engagement [61]. Health consequences of stressors depend upon different types and amount of resources available in a community, such as coping, supports and trust [62, 63]. Numerous studies have explored the buffering role of social support as community resources in attenuating negative consequences of external stressors; however less is known about the role and the function of social capital in the stress-buffering process [64]. Therefore, future research is needed to examine underlying mechanisms through which social capital can function as a buffer for the malicious effects of stressors on those who are socially or physically disadvantaged.

The findings also suggest that strong perceptions about communities (i.e., perceived community cohesion) would enable individuals to improve health and well-being of community members [29]. Recent studies on social capital have indicated that a higher level of perceived cohesion was associated with better mental health [65, 66]. Consistent with prior evidence, we found the direct effect of the cognitive component of social capital (perceived cohesion) on mental health status, but not on physical health status. A possible explanation is that the pathways from social capital and social relations to mental health are shorter than the pathways to physical health [43, 67]. This is an area of research that requires further investigation on the effects of perceptions about communities on physical health by testing potential mediators and moderators.

In addition to the effects of social capital, our findings suggest that perceived aspects of neighbourhood climate may be an important determinant of community health and well-being. Previous studies [68–70] have extensively studied the effects of neighbourhood environmental characteristics on various health outcomes, such as life satisfactions and psychological distress. In this study, we found that perceptions of poor quality of neighbourhood environment contributed to worsen mental health among Australian adults. In this regard, building a high quality neighbourhood climate is important to enhance positive perceptions about neighbours and neighbourhoods, which would in turn deliver favorable health and social outcomes. Future interventions may involve local councils and grassroots community organisations to explore and evaluate changes to the composition of the community and neighbourhood settings.

### Limitations

We note some limitations in this study. First, the current study focused on individual-level social capital and did not take into account the influence of ecological and cross-level social capital (e.g., geographic variations in the level of social capital). Future research may utilise a nested study design with clusters of neighbourhoods or communities to assess the impact of the aggregated-level social capital on health related outcomes. Second, there is another line of social capital research that examines social connections and resources emerged within (homogeneity) and between (heterogeneity) groups or communities, namely bonding and bridging social capital [9]. Although this investigation was beyond the scope of the paper, we believe that it is particularly important to explore how different levels or types of social ties/relations (within and between groups/communities) could influence health in multi-cultural and multi-ethnic countries including Australia. Third, this study utilised the two norm-based scores of SF-36 as the primary outcome variables (i.e., PCS and MCS). Given the significant relationships between social capital and mental health, further analyses with the subscales of mental health status (e.g., vitality, role-emotional) would be a useful avenue for future research. Finally, study results may not be generalizable to those persons with illness conditions and disabilities that render them unable to be interviewed and persons with English language difficulties.

In spite of these limitations, findings from this study remain useful in the design of effective community interventions and social policies for the promotion of positive health and behavioural outcomes. In particular, fostering community participation (structural social capital) could be a promising intervention strategy to facilitate better health outcomes in community or neighbourhood settings by attenuating negative consequences of stressors among those with chronic health conditions, impairments or disabilities.

## Conclusions

This study investigated the differential effects of the structural and cognitive social capital on the two forms of health status (physical and mental health) among Australian adults. One of the most notable findings is that community participation (structural social capital) moderated the association between chronic health conditions and mental health status. This suggested that promoting the structural social capital would function as a buffer against the malicious effects of chronic health conditions and disabilities. Specifically, community participation is indispensable to develop an effective community-based program to improve health and well-being of those with chronic health conditions or disabilities, as increasing active participation may generate beneficial effects in this vulnerable population. Subjective perceptions about communities are also a significant factor to improve health outcomes. Consistent with our anticipations, the study found that perceived cohesion was predictive of mental health status. Further research is needed to examine underlying mechanisms linking the multiple dimensions of social capital to health outcomes among individuals who are vulnerable to external stressors.

## Data Availability

The data (HILDA Survey General Release) that support the findings of this study are available from the Australian Data Archive (ADA) but restrictions apply to the availability of these data, which were used under license for the study, and so are not publicly available.

## Abbreviations

HILDA Survey: Household, Income and Labour Dynamics in Australia Survey
MCS: Mental component score
PCS: Physical component score

## References

[1] Cohen S (2004). Social relationships and health. Am Psychol.

[2] Kawachi I, Berkman L (2000). Social cohesion, social capital, and health. Soc Epidemiol.

[3] Kawachi I, Subramanian IV, Kim D (2008). Social capital and health. Social Capital and Health.

[4] Murayama H, Fujiwara Y, Kawachi I (2012). Social capital and health: a review of prospective multilevel studies. J Epidemiol.

[5] Rocco L, Suhrcke M (2012). Is social capital good for health? A European perspective. Euro Who Int.

[6] Farr J (2004). Social capital: a conceptual history. Political Theory.

[7] Putnam RD (2000). Bowling alone: The collapse and revival of American community.

[8] Harpham T (2002). Measuring social capital within health surveys: key issues. Health Policy Plan.

[9] Almedom AM (2005). Social capital and mental health: an interdisciplinary review of primary evidence. Soc Sci Med.

[10] Berry H, Rickwood D (2000). Measuring social Capital at the Individual Level: personal social capital, values and psychological distress. J Public Ment Health.

[11] Berry HL, Welsh JA (2010). Social capital and health in Australia: an overview from the household, income and labour dynamics in Australia survey. Soc Sci Med.

[12] Ding N, Berry HL, O’Brien LV (2015). One-year reciprocal relationship between community participation and mental wellbeing in Australia: a panel analysis. Soc Sci Med.

[13] Shipley M, Berry HL. Longing to Belong: Personal Social Capital and Psychological Distress in an Australian Coastal Region. FaHCSIA Soc Policy Res Pap No 39. 2010; [Cited 2019 Sep 23]; Available from: http://www.ssrn.com/abstract=1703238.

[14] Whitley R, McKenzie K (2005). Social capital and psychiatry: review of the literature. Harvard Rev Psychiatry.

[15] Berry HL, Rodgers B, Dear KBG (2007). Preliminary development and validation of an Australian community participation questionnaire: types of participation and associations with distress in a coastal community. Soc Sci Med.

[16] Kawachi I, Kennedy BP, Glass R (1999). Social capital and self-rated health: a contextual analysis. Am J Public Health.

[17] Maass R, Kloeckner CA, Lindstrøm B, Lillefjell M (2016). The impact of neighborhood social capital on life satisfaction and self-rated health: a possible pathway for health promotion?. Health Place.

[18] Poortinga W (2006). Social relations or social capital? Individual and community health effects of bonding social capital. Soc Sci Med.

[19] Kawachi I, Berkman LF (2001). Social ties and mental health. J Urban Health.

[20] Kawachi I, Kennedy BP, Lochner K, Prothrow-Stith D (1997). Social capital, income inequality, and mortality. Am J Public Health.

[21] Lindström M, Hanson BS, Wirfält E, Östergren PO (2001). Socioeconomic differences in the consumption of vegetables, fruit and fruit juices: The influence of psychosocial factors. Eur J Pub Health.

[22] Addy CL, Wilson DK, Kirtland KA, Ainsworth BE, Sharpe P, Kimsey D (2004). Associations of perceived social and physical environmental supports with physical activity and walking behavior. Am J Public Health.

[23] Giordano GN, Lindström M (2011). The impact of social capital on changes in smoking behaviour: a longitudinal cohort study. Eur J Pub Health.

[24] Lindström M, Giordano GN. Changes in social capital and cigarette smoking behavior over time: a population-based panel study of temporal relationships. Nicotine Tob Res. 2016;18(11):2106–2114. Available from: https://academic.oup.com/ntr/article-lookup/doi/10.1093/ntr/ntw120. 10.1093/ntr/ntw120 27113013

[25] Lindström M, Moghaddassi M, Merlo J, Bolin K, Lindgren B (2003). Social participation, social capital and daily tobacco smoking: a population-based multilevel analysis in Malmö, Sweden. Scand J Public Health.

[26] Rogers EM (2003). Diffusion of innovations.

[27] Alesina A, La Ferrara E (2000). Participation in heterogeneous communities. Q J Econ.

[28] Kawachi I, Berkman LF. Neighborhoods and Health: New York: Oxford University Press; 2009. p. 1–352.

[29] Yamaguchi A (2013). Influences of social capital on health and well-being from qualitative approach. Global J Health Sci.

[30] Walker RB, Hiller JE (2007). Places and health: a qualitative study to explore how older women living alone perceive the social and physical dimensions of their neighbourhoods. Soc Sci Med.

[31] Wilkinson RG. Unhealthy societies: The afflictions of inequality. Routledge; 2002. Available from: https://www.taylorfrancis.com/books/9780203421680.

[32] Kim ES, Kawachi I (2017). Perceived neighborhood social cohesion and preventive healthcare use. Am J Prev Med.

[33] Echeverría S, Diez-Roux AV, Shea S, Borrell LN, Jackson S (2008). Associations of neighborhood problems and neighborhood social cohesion with mental health and health behaviors: The multi-ethnic study of atherosclerosis. Health Place.

[34] Diez-Roux AV, Mujahid MS, Hirsch JA, Moore K, Moore LV (2016). The impact of neighborhoods on cardiovascular risk: the MESA neighborhood study. Lancet Glob Health.

[35] Australian Institute of Health and Welfare (2019). Chronic disease Overview - Australian Institute of Health and Welfare.

[36] Paez KA, Zhao L, Hwang W (2009). Rising out-of-pocket spending for chronic conditions: a ten-year trend. Health Aff.

[37] Islam MM, Valderas JM, Yen L, Dawda P, Jowsey T, McRae IS (2014). Multimorbidity and comorbidity of chronic diseases among the senior australians: prevalence and patterns. PLoS One.

[38] Megari K (2013). Quality of life in chronic disease patients. Health Psychol Res.

[39] Vancampfort D, Stubbs B, Koyanagi A (2017). Physical chronic conditions, multimorbidity and sedentary behavior amongst middle-aged and older adults in six low- and middle-income countries. Int J Behav Nutr Phys Act.

[40] Campbell C, McLean C (2002). Ethnic identities, social capital and health inequalities: factors shaping African-Caribbean participation in local community networks in the UK. Soc Sci Med.

[41] Fitzpatrick KM, Wright DR, Piko BF, Lagory M (2005). Depressive symptomatology, exposure to violence, and the role of social capital among African American adolescents. Am J Orthop.

[42] Mandelbaum J, Moore S, Silveira PP, Meaney MJ, Levitan RD, Dubé L (2018). Does social capital moderate the association between children’s emotional overeating and parental stress? A cross-sectional study of the stress-buffering hypothesis in a sample of mother-child dyads. Soc Sci Med.

[43] Klijs B, Mendes de Leon CF, EUB K, Smidt N (2017). Do social relations buffer the effect of neighborhood deprivation on health-related quality of life? Results from the LifeLines Cohort Study. Health Place.

[44] Mehbub Anwar AHM, Astell-Burt T, Feng X (2019). Does social capital and a healthier lifestyle increase mental health resilience to disability acquisition? Group-based discrete trajectory mixture models of pre-post longitudinal data. Soc Sci Med.

[45] Beaney T, Burrell LM, Castillo RR, Charchar FJ, Cro S, Damasceno A (2019). May measurement month 2018: a pragmatic global screening campaign to raise awareness of blood pressure by the international society of hypertension. Eur Heart J.

[46] Rashid AA, Devaraj NK (2018). Oh no! now i have diabetes. Rawal Med J.

[47] Chia Y-C, Ching SM, Chew BN, Devaraj NK, Siti Suhaila MY, Tay CL (2019). May measurement month 2017 blood pressure screening: findings from Malaysia—South-East Asia and Australasia. Eur Hear J Suppl.

[48] Watson N (2012). M W. The HILDA survey: a case study in the design and development of a successful household panel survey. Longit Life Course Stud.

[49] Wilkins R, Lass I (2018). The household, income and labour dynamics in Australia survey: selected findings from waves 1 to 16.

[50] Summerfield M, Bevitt A, Freidin S, Hahn M, La N, Macalalad N, O’Shea M, Watson N, Wilkins R, Wooden M (2017). HILDA user manual – release 16.

[51] Shields MA, Wheatley Price S, Wooden M (2009). Life satisfaction and the economic and social characteristics of neighbourhoods. J Popul Econ.

[52] Stafford M, Stansfeld S, Shipley M, Marmot M, Hemingway H (1997). Is the SF-36 a valid measure of change in population health? Results from the Whitehall II study. BMJ.

[53] Gandek B, Sinclair SJ, Kosinski M, Ware JE (2004). Psychometric evaluation of the SF-36® health survey in medicare managed care. Health Care Financ Rev.

[54] Australian Bureau of Statistics (1995). National Health Survey: SF36 Population Norms, Australia.

[55] Australian Bureau of Statistics. Australian Health Survey: Updated Results, 2011-2012. [Cited 2020 Jan 23]. Available from: https://www.abs.gov.au/ausstats/abs@.nsf/Lookup/4364.0.55.003Chapter12011-2012.

[56] Muthén L, Muthén B. Mplus user’s guide (6th ed). Los Angeles; 2012.

[57] Schafer JL, Graham JW (2002). Missing data: our view of the state of the art. Psychol Methods.

[58] Graham JW, Cumsille PE, Elek-Fisk E, Schinka JA, Velicer WF (2003). Research methods in psychology. Methods of handling missing data.

[59] Pearlin LI (1999). The stress process revisited: reflections on concepts and their interrelationships. Handbook of the Sociology of Mental Health.

[60] Thoits PA (2010). Stress and Health: major findings and policy implications. J Health Soc Behav.

[61] Haldane V, Chuah FLH, Srivastava A, Singh SR, Koh GCH, Seng CK (2019). Community participation in health services development, implementation, and evaluation: A systematic review of empowerment, health, community, and process outcomes. Maulsby C, editor. PLoS One.

[62] Vassilev I, Band R, Kennedy A, James E, Rogers A (2019). The role of collective efficacy in long-term condition management: a metasynthesis. Health Social Care Community.

[63] Hibino Y, Takaki J, Ogino K, Kambayashi Y, Hitomi Y, Shibata A (2012). The relationship between social capital and self-rated health in a Japanese population: a multilevel analysis. Environ Health Prev Med.

[64] Saegert S, Carpiano RM (2017). Social support and social capital: A theoretical synthesis using community psychology and community sociology approaches. APA handbook of community psychology: Theoretical foundations, core concepts, and emerging challenges.

[65] Berry H, Shipley M (2011). Longing to belong: Social capital and mental health in an Australian coastal community.

[66] Bassett E, Moore S (2013). Mental Health and social capital: social capital as a promising initiative to improving the mental Health of communities. Current Topics in Public Health.

[67] Berkman LF, Glass T, Brissette I, Seeman TE (2000). From social integration to health: Durkheim in the new millennium. Soc Sci Med.

[68] Eibich P, Krekel C, Demuth I, Wagner GG (2016). Associations between neighborhood characteristics, well-being and health vary over the life course. Gerontology.

[69] Brown SC, Mason CA, Spokane AR, Cruza-Guet MC, Lopez B, Szapocznik J (2009). The relationship of neighborhood climate to perceived social support and mental health in older hispanic immigrants in miami, florida. J Aging Health.

[70] Shields M, Wooden M (2003). Investigating the role of neighbourhood characteristics in determining life satisfaction.

